# A Systems Dynamic Model for Drug Abuse and Drug-Related Crime in the Western Cape Province of South Africa

**DOI:** 10.1155/2017/4074197

**Published:** 2017-05-07

**Authors:** Farai Nyabadza, Lezanie Coetzee

**Affiliations:** Department of Mathematical Sciences, Stellenbosch University, Private Bag X1, Matieland 7600, South Africa

## Abstract

The complex problem of drug abuse and drug-related crimes in communities in the Western Cape province cannot be studied in isolation but through the system they are embedded in. In this paper, a theoretical model to evaluate the syndemic of substance abuse and drug-related crimes within the Western Cape province of South Africa is constructed and explored. The dynamics of drug abuse and drug-related crimes within the Western Cape are simulated using STELLA software. The simulation results are consistent with the data from SACENDU and CrimeStats SA, highlighting the usefulness of such a model in designing and planning interventions to combat substance abuse and its related problems.

## 1. Introduction

The complex problems that society and researchers face in current times leave no space for one-dimensional thinking with regard to interventions. Community level problems cannot be viewed in isolation. In fact, they need to be viewed within the system they are embedded in. Substance abuse is one of the complex challenges that researchers in different fields are battling to understand and analyse, especially with regard to the ripple effects it has within communities in terms of drug-related crimes and the spread of sexual transmitted infections. Systems thinking and system dynamics open up new avenues for cross-discipline research. This enables the social sciences and natural sciences to integrate descriptive and quantitative research in order to inform communities and assist stakeholders and policy makers at the same time.

South Africa's geographic location, lax border controls, a weak criminal justice system, modern telecommunications, banking systems, and international trade links with South America, North America, Asia, and Europe endanger the country to transhipment of drugs [1]. Even though substance abuse is a national menace in South Africa, it is found to be concentrated within the Western Cape [2–4]. According to the Department of Community Safety [2], more than a third (35%) of the crimes in the Western Cape are due to substance abuse. This explains the high contribution the Western Cape has had on the national drug-related crime statistics in the past decade (consistently higher that 30%). In the 2013/2014 report on the Western Cape policing needs and priorities (PNP), it was noted that substance abuse is the motivation behind 22% of committed crimes [2].

The dynamic evolution of drug abuse within communities is synonymous to that of infectious diseases; see, for instance [5–9] and the references cited therein, where initiation of the susceptible population is dependent on interaction between the drug users and the population at risk. White and Gorman [10] explained the complex relationship between drugs and crime through three models: “(1) substance use leads to crime, (2) crime leads to substance use, and (3) the relationship is either coincidental or explained by a set of common causes.” These models explain the different reasons why crimes are being committed by drug-using offenders. Although a single model cannot explain the entire drug-crime system, the relationship between drugs and crime involves a broad spectrum of social, political, and economic forces, the environment of the individuals abusing substances, and the biological processes driving human behaviour [11].

In 2004, 4563 addicts were rehabilitated in the Western Cape represented by data collected in the Cape Town area. In 2013, 7195 addicts were rehabilitated across the WC, indicating a 63.4% increase in admissions [12]. A 311.5% increase in drug-related crimes was also observed since the 2003/2004 annual report of the SAPS [13]. Annual drug-related arrests increased from 19,940 cases in 2003/2004 to 82,062 cases in 2012/2013 [4]. The increased prevalence of crimes associated with substance abuse underscores the importance of examining the system of interacting processes and feedback loops that are associated with substance abuse and drug-related crimes. It is also imperative to first take into account what the local culture, context, and politics are when considering a system's solution [14].

This paper focuses on the occurrences of substance abuse and drug-related crimes in the Western Cape during the time period 2004–2014. This study explores the current state of drug abuse and its relation to drug-related crimes within the Western Cape in South Africa from a systems thinking and systems dynamic perspective. The focus is on gaining a better understanding of the underlining structure of the system that sustain drug abuse and drug-related crimes within the communities, as well as exploring the relationship between the two, within the system. The aim of this paper is thus to investigate the relationship between substance abuse and drug-related crimes within the Western Cape. A dynamic system is constructed on the STELLA platform in order to explore and understand the relationships and structures within the substance abuse and drug-related crime system. The model is linked to data from the South African Community Epidemiology Network on Drug Use (SACENDU), Crime Stats SA, Census South Africa, and SA health info, for the simulations.

## 2. Systems Thinking, System Dynamics, and STELLA

Systems thinking focuses on studying the whole, by employing functional and relational principles [15–17], rather than focusing on the simple elements as preferred in reductionistic mathematical models such as the* SIR* models. Interrelatedness and emergence are some of the fundamental ideas that systems thinking is built on [18], where an emergent property of a system is a product of the system as a whole. Systems thinking allows us to consider the product of the system and not only that of a single component in the system.

Conceptual models constructed from the perspective of systems thinking allow researchers to interpret or explain social phenomena by relating different structures that shape our lives like biological, organizational, political, and governmental systems [15–17, 19, 20]. Systems thinking requires critical thinking of researchers in various fields and holds the possibility of exposing assumptions that are systemically flawed in mental and mathematical models that researchers have often based their work on.

Systems thinking and system dynamics bring a new perspective and opportunity for active engagement and trans-disciplinary thinking with those who have stake in the outcome to govern the course of change. This is done in order to consider connections of different components and plan for the implications of their interaction [15–17]. A common theme within systems is their dynamic nature, whether equilibriums, cycles, or chaotic behaviour occurs. Dynamic change is always present within systems. Complexity of a system rises out of the multiple stakeholders that often have different and/or competing goals [20]. This phenomenon in systems causes agents to constantly adapt within the system, forming a self-organizing system that is sensitive to preexisting conditions and governed by both the actions and reactions of the system actors [3, 15, 16]. Holder [14] recommended health research to use systems principles and orientation, when approaching health hazards such as substance abuse as a system of structured relationships with diverse methodologies. Systems research creates the opportunity to critically explore what hinders or accelerates adoption of evidence based strategies and promote their implementation [18]. System dynamics assist in understanding how these systems are organized and sustained and discover the possible ways in which they can be improved within their democratic and dynamic environments.

In summary, dynamic systems consist of a trans-disciplinary integration that aims to reconcile linear and nonlinear, qualitative and quantitative, reductionist, and holistic thinking and methods into union [14]. Leischow et al. [16] explained how most causations of diseases are nonlinear, dynamic, and multifactorial. Systems thinking reflects both the functional and conceptual areas of substance abuse, as well as the burden it has on its environment, namely, drug-related crime. Swanson et al. [17] stated that even though systems are not a recent concept, “adaptive systems present novel opportunities for synergy and increasing capacity in local communities and organizations.”

STELLA has* stocks*,* flows*,* converters,* and* connectors* used to visually construct a model to represent the system. Relationships within the system are depicted by* stocks* that are increased and decreased by* flows*, and the* flows* are governed by* converters* and* connectors*. A* stock* accumulates over time and is altered through* flows* (increased by an inflow and decreased by an outflow). Thus, a stock is the representation of the net flow at a specific point in time. Stocks and flows are, mathematically speaking, differential and integral equations. The flow between the many interrelated parts in the system is governed by feedback loops. There are two types of feedback loop: negative feedback loops known also as balancing feedback loop and positive feedback loops known as amplifying/reinforcing loops [15]. Balancing loops assist the system in reestablishing standard conditions, unlike the reinforcing loops that lead to the growth of a trend. The interplay of these different loops leads to steady states, where the emergent whole at the end result is known as a finite one.

## 3. Methodology

### 3.1. Model Formulation

#### 3.1.1. Model and Its Boundaries

This model is based on the premised idea that a community is an interacting set of systems that support or buffer the occurrence of certain dynamics, such as substance abuse and drug-related crimes. The systemic approach to the model firstly enables us to create a system model that can capture the primary community structures and relationships (stocks and flows) that sustain substance abuse and drug-related crimes within the community. Secondly, it allows us to critically test plausible strategies to reduce or counteract the problem of drug abuse.

The model is derived from empirical evidence and literature since the research available concerning key stocks and flows in this model is limited and there are few published studies on the topic available, especially with regard to the Western Cape. We also make some informed assumptions based on what is currently known. In this study, the system constructed is not a single organizational entity but rather an integrated and interacting community response to substance abuse and drug-related crime, through a consideration that looks at the rehabilitation processes of both cases.

The constructed model is named the substance abuse and drug-related crime in the Western Cape (SADC-WC) model, for the estimation and prediction of connections between substance abuse and drug-related crime within communities in the Western Cape. The boundary of this model is the total area of the Western Cape province which constitutes one metropolitan municipality, namely, the city of Cape Town, and five district municipalities, namely, Cape Winelands, Central Karoo, Eden, Overberg, and West Coast. The five district municipalities beset 24 local municipalities. Within the geographical boundaries there are 150 police precincts, 60 precincts are classified as urban because they fall within the Cape Metropolitan area and 90 police precincts are classified as rural as they fall outside the city of Cape Town [2]. The model boundaries also include 32 specialist treatment centres/programs that participate in the SACENDU project in the Western Cape [12, 21].

#### 3.1.2. Stocks and Flows

The SADC-WC system is constituted of the stocks and flows listed in Table 1. The broader susceptible community (individuals at risk) are represented by the *C* stock. Individuals abusing any form of substance or taking part in any form of drug-related crime (possession, trading, use, or motivation for crime) are in stock *U*. Even though the responsibility of safety is webbed within the community between different stakeholders, the South African Police Services (SAPS) are responsible for preventing, combating, and investigating crime. Therefore the police enforcement is a stakeholder within our system, which is represented by drug-related crime cases made by the SAPS in stock *L*. In this paper the levels of functionality of SAPS are measured by the changes in the number of cases recorded annually. All the cases that are successfully convicted are represented by the correctional service stock *S*. The specialist treatment and rehabilitation centres are depicted by *R*, the stock for individuals that are undergoing rehabilitation, whether by own accord or through the recommendation of correctional services.

#### 3.1.3. Sources of Information for Flows

We now consider what informs the flows in Table 1. The community is governed by an inflow that is dependent on births and net migration and an outflow dependent on deaths. The Western Cape has an estimated population of 5.82 million people according to Statistics South Africa [22], displaying an increase in the population since 2004 that could be attributed to labour migration, since people in South Africa tend to leave their provinces or usual residences in search for work in industrialised provinces such as the Western Cape. Initiation of individuals within the community at risk into drug use and drug-related crimes is caused when individuals from *C* and *D* interact. Self-conviction for early substance users or drug-related crime is taken into account as a flow from *D* to *C* and is often governed by the self-efficacy of the individual. The White and Gorman [10] models that study the complex relationship of drugs and crime assume that substance use can cause drug-related crime and similarly drug-related crime can lead to substance use. Therefore, the *D* stock consists of both substance users and criminals that commit drug-related offences. Most crimes reported by the SAPS do not make it to the court and are represented within the failed convictions flow. The flows influencing stock *L* are based on the progress and finalisation of cases. The conviction flow feeding *S* is thus based on the successful conviction flow out of *L*. Individuals within *S* can be referred to *R* for rehabilitation or complete their sentence time and move back into the community *C*.

Connectors exhibit how one variable causes another variable to change, indicating cause and effect within the system. The connectors (parameters) of the SADC-WC system are listed in Table 4. Data that is relevant to internal linkages within the system is gathered from the multiple data sources available to support the flows and conditions within and among the stocks in this system. The connectors that implicate the uptake of individuals into *R* were based on the data collected from SACENDU [21, 23]. The birth and death rate values are supported by the census data of 2004 and 2011 [22, 24]. Relapse and successful rehabilitation rates are supported by the proportion of first-time patients admitted to rehabilitations in the latest reports of SACENDU for the Western Cape rehabilitation centres [21, 25]. The arrest and conviction rates are based on information obtained from the SAPS and Crime Stats SA [3, 4].

### 3.2. Model Diagram and Equations That Determine Flows

**Figure 1:**
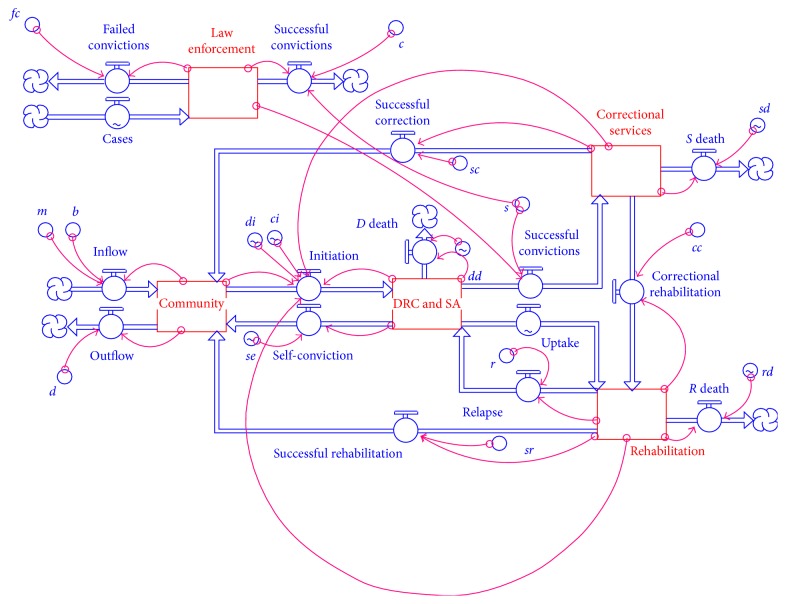
The SADC-WC system in STELLA.

Converters either hold a constant value or apply an equation and convert a set of inputs into outputs. Thus converters are the flows which if not constant are mathematical equations that link the stocks to each other. All the converters in the SADC-WC model are listed in Table 2. The schematic representation of the model is shown in Figure 1.

The SADC-WC stocks are governed by the following differential equations:

(1) dCdt=Inflow+Successful  rehabilitation+Successful  corrections+Self  convictions−Outflow−Initiation,dDdt=Initiation+Relapse−Uptake−Successful  convictions−Self  convictions−Drug  and  crime  related  deaths,dSdt=Successful  convictions−Correctional  rehabilitation−Successful  corrections−Correctional  service  related  deaths,dRdt=Uptake+Correctional  rehabilitations−Successful  rehabilitation−Relapse−Rehabilitaion  related  deaths,dLdt=Cases−Failed  convictions−Successful  convictions.

When one considers the first equation, the inflow is the sum of the births (with a birth rate *b*) and the net immigration, *m*. This is represented by the term *b∗C* + *m*, where *∗* represents multiplication of the parameter and the state variable. The remaining part of the first equation and the entire system are presented in Appendix.

## 4. Simulations and Results

### 4.1. Initial Conditions Estimation

The initial values for the community, drug-related crimes, and rehabilitation stocks were derived from the data sources in [3, 12, 22, 24]. The drug-related crimes for the WC were recorded as 30432 cases [26]; thus the initial number of drug-related crime cases *L*(0) = 30432 in 2004. According to the SACENDU's report [12], 4563 patients were treated in 2004 in rehabilitation centres in the WC, so we take *R*(0) = 4563. The total estimated population for the WC in 2004 are 4645600 [27]. According to Health 24, the substance abusing population can be estimated at 15% of the total population [28]; thus *U*_0_ = 696840. The initial value of *S*(0) was estimated based on the conviction rate and the initial value for *L* [23, 26]. The initial conditions are summarised in Table 3.

### 4.2. Parameter Estimation

Parameters within a STELLA model are modelled as constant values in converters. The parameters used within the SADC-WC system, in Table 4, are values gathered from data sources as those referenced or, in the instances where data was not available, values were obtained from literature; see the cited references and the known dynamics of the model. Assumed parameters were modified iteratively till resulting changes in the behaviour of the system aligned with expectations of the model simulations which were guided by data available. This form of hit and miss is necessary in areas where extensive research data is not available.

The birth and death rates for the system are based on the average values for South Africa during the 2004–2014 period [26]. The movement of people in South Africa due to labour migration motivated the addition of migration into the model. The estimated WC migration streams for 2004–2014 averaged on a net migration of 152230 people; we therefore assume an average inflow of 152230 individuals (assumed at risk) into the SADC-WC system annually. The relapse ratio and successful rehabilitation values are based on the average percentages of first time admissions in SACENDU's reports for the ten-year period [21, 29, 30] correctional service referral to *R*; namely, *cc* is estimated as an average for the 2004–2014 period [21, 29, 30]. The cases making it to court, cases not making it to court, and successful conviction rates *c*, *fc*, and *s*, respectively, are estimated according to the research on conviction rates of all crime categories by Leggett [31], which are based on the Law Commission report on conviction rates and other outcomes of crimes reported in eight South African police areas [30]. It is indicated in the report that, of 25% of cases that make it to the court, only a quarter produce convictions within the period of investigation [31, 32]. The uptake rate *u* of individuals into *R* is based on the percentage of growth of *R* for the 2004–2014 period [23]. Successful corrections are estimated as a function *τ*, which is dependent on the average duration of offenders in *S* that are sentenced as mentioned in Section 1. According to the Department of Correctional Services, 31% of offenders are sentenced for 1 month to 5 years, 42% for 5 to 15 years, 10% for 15 to 20 years, and 16% for 20 years and longer [33]. According to these findings, the median of the respective times spent in *S* was used to determine the flow of successful corrections, where

(2) sc12.5∗0.31+110∗0.42+112.5∗0.1+130∗0.16=0.179.

The drug and crime initiation and self-conviction values are estimated to be in the range of 0.01–0.05. Finally, the deaths occurring within the *D*, *R*, and *S* stocks are assumed to be higher than those of the susceptible population in *C* and are in the range of 0.015–0.02.

### 4.3. SADC-WC System Simulations

**Figure 2:**
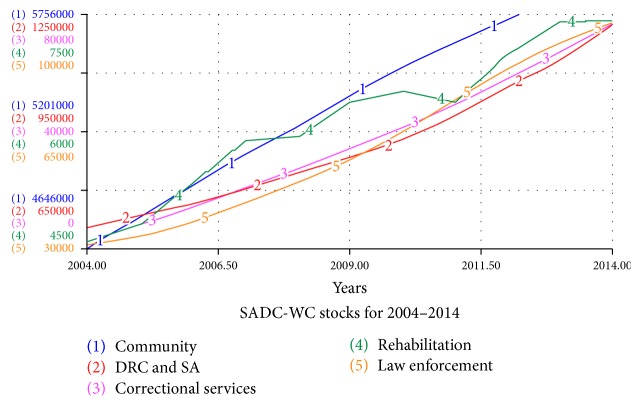
The stocks of the SADC-WC system for 2004–2014.

**Figure 3:**
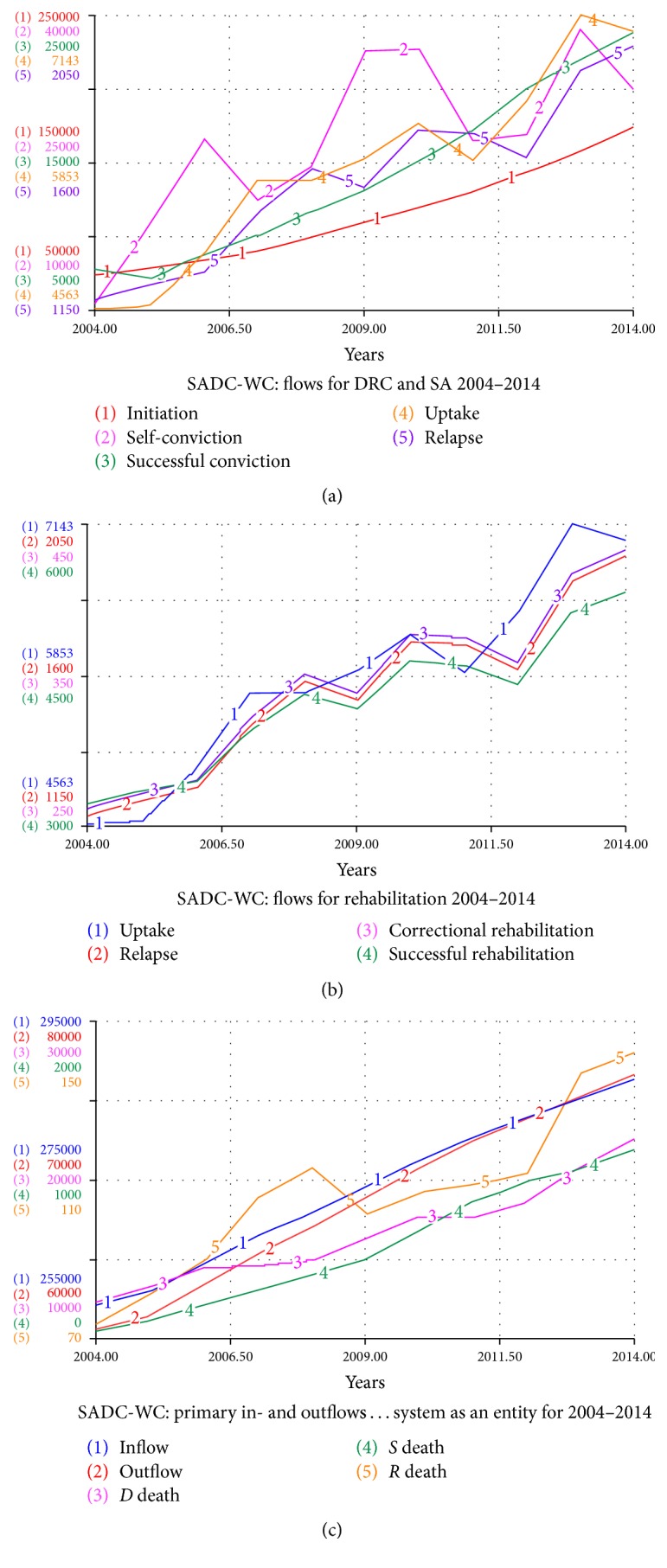
The flows of the SADC-WC system for 2004–2014.

The simulations of the SADC-WC system's stocks and flows for 2004–2014 are illustrated in Figures 2 and 3, respectively. An important feature of the SADC-WC system is the inclusion of law enforcement as a stock. This enables us to use the known data from the SAPS of drug-related cases made per year in the Western Cape. Therefore, we consider the successful conviction flow to depict the similar flows to those that are currently occurring in the Western Cape. Since the model is fitted to the data, we assume that the inflows and outflows of the law enforcement and correctional service are an adequate depiction of what is currently happening within the criminal justice system of the Western Cape. The law enforcement stock in Figure 2 indicates that the cases of drug-related crime are ten times more than successful convictions. This suggests the failure of the criminal justice system to convert cases made into successful convictions. This is often due to cases not making it to court and the high numbers of failed convictions.

In Figure 2 we also notice that the law enforcement, correctional service, and drug abuse and drug-related crime increased exponentially over the past decade. We deduce that substance abuse and drug-related crime are close to ten times more than the current cases that are reported by the law enforcement. This may highlight the extent of the substance abuse and drug-related crime in the Western Cape.

Figure 3 illustrates the inflows and outflows for each stock in the SADC-WC system for the 2004–2014 period. We notice that the successful inflow from correctional services into the community is significantly higher than successful rehabilitations. This highlights the fact that a limited capacity of rehabilitation centres is a limit to the substance abuse → rehabilitation → community loop. We also notice that the successful convictions flow is significantly higher than the uptake flow into rehabilitation in Figure 3(a), again mirroring the restraint of limited space in rehabilitation. An unexpected observation in Figure 3(b) is how little the contribution of correctional service referrals to rehabilitation is. Finally, Figure 3(c) allows us to compare the deaths occurring in the Western Cape. We notice that the deaths due to drug abuse and drug-related crimes are significantly high, indicating the burden the syndemic has on communities.

### 4.4. SADC-WC System Projected Simulations

The SADC-WC system is used to simulate the expected scenarios in the next five years of the syndemic currently taking place in the Western Cape, if it persists as it currently is. Thus, projected simulations are done for the period 2004–2019. It is assumed that the increase in uptake into rehabilitation will be similar to the average growth in uptake over the period of 2004–2014 [23]. It is also so assumed that drug-related crime cases will show similar growth as in the previous five years according to data from the SAPS [4].

**Figure 4:**
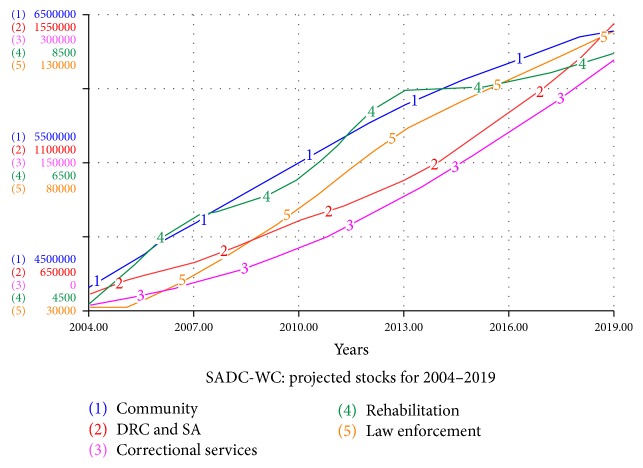
Projected stocks and flows for the SADC-WC system for 2004–2019.

From the projected simulations in Figure 4, we see that drug abuse and drug-related crime will increase by 50% in the next five years, if action is not taken to contain or reduce the syndemic. The rehabilitation stock only grew from 4000 to 8500, highlighting the limited capacity of rehabilitation centres in the Western Cape. It is interesting that the projections indicate that the substance abuse and drug-related crime stock, as well as the correctional service stock, will grow exponentially in the next five years. The correctional service referrals to rehabilitation are still expected to remain very low in the next five years.

### 4.5. Interventions

We now consider the institution of interventions and project the possible outcomes of three interventions that can possibly be instituted in the fight against this syndemic.

#### 4.5.1. Intervention  1: Effect of Increased Convictions

**Figure 5:**
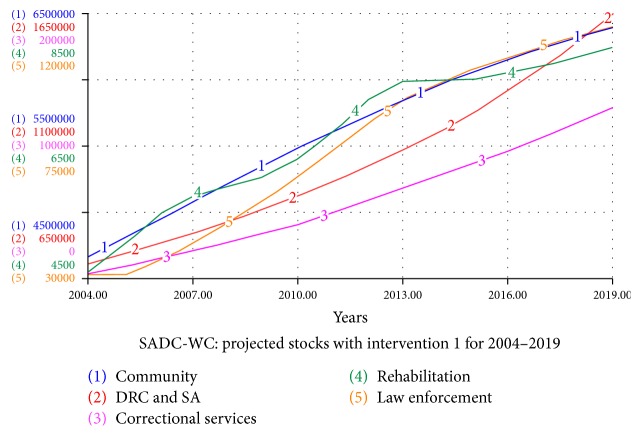
Projected stocks for the SADC-WC system with intervention 1 for 2004–2019.

The effects of increasing cases going to court and successful convictions as an intervention are investigated. The SADC-WC system is studied assuming that the cases making it to court and successful convictions will double over the next five years. Both *c* and *s* are increased linearly over a period of five years (2014–2019) from 0.25 to 0.5 to investigate how this will affect the system as a whole and how it compares with the other interventions. In Figure 5 the simulations for the different stocks and flows are shown.

We notice that increase of cases going to court and successful conviction rate has a significant impact on the syndemic. We see that drug-related crime cases made by the law enforcement show a decrease towards 2019, where as in Figure 2 we observe an almost continuous linear growth of the law enforcement stock.

#### 4.5.2. Intervention 2: Effect of Increased Correctional Service Referrals to Rehabilitation Centres

The effects of increasing the rate at which correctional services refer individuals to rehabilitation centres as an intervention are investigated. The system is studied assuming that the rate at which correctional services refer individuals to rehabilitation centres over the next five years is doubled. Thus, *cc* is increased linearly over a period of five years (2014–2019) from 0.05 to 0.10.

**Figure 6:**
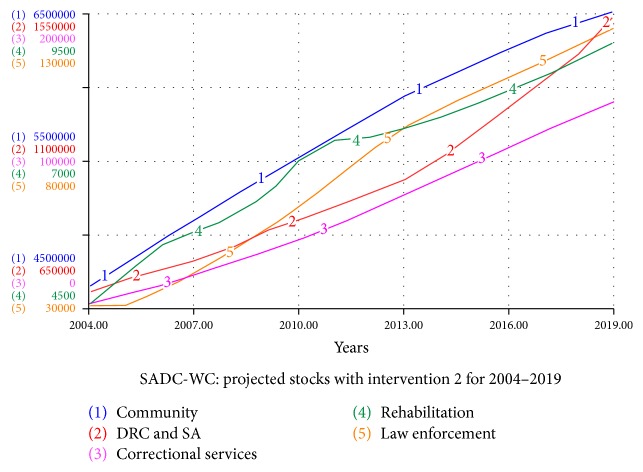
Projected stocks for the SADC-WC system with intervention 2 for 2004–2019.

The flow of referrals from correctional services to rehabilitation centres is very low currently in the Western Cape. We notice from the simulations in Figure 6 that even if the rate at which convicted criminals with a substance abuse addiction are referred to rehabilitation is doubled, it will not have a significant impact on the SADC-WC system as a whole. We note that the rehabilitation stock does not increase significantly in Figure 6, in comparison to Figure 2 where no interventions are taken into consideration.

#### 4.5.3. Intervention 3: The Effect of Reducing Relapsing

**Figure 7:**
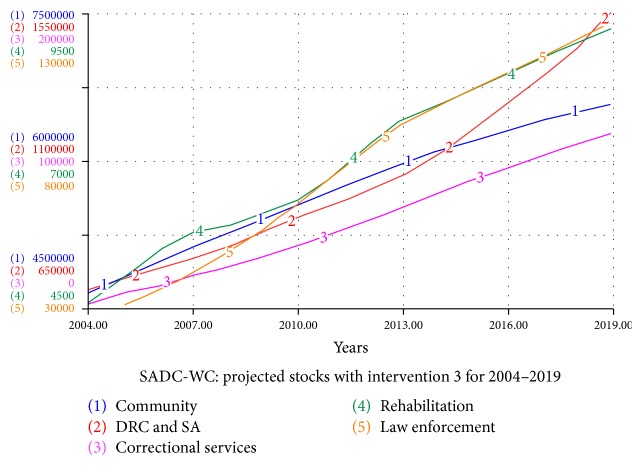
Projected stocks for the SADC-WC system with intervention 3 for 2004–2019.

The effects of decreasing the percentage of individuals that relapse back into substance abuse and drug-related crime as an intervention are investigated, and the results are displayed in Figure 7. The system is studied assuming that there will be a decrease in the percentage of individuals that relapse back into substance abuse and drug-related crime (*D*) over the next five years. This can be done by increasing the efficiency of rehabilitation programs or increasing the duration of rehabilitation programs. Thus, *R* is decreased linearly over a period of five years (2014–2019) from 0.257 to 0.127.

The relapse inflow (depicted by the flows) into drug abuse and drug-related crimes falls drastically, but still the drug abuse and drug-related crime stock are very similar to those in Figure 2. This again suggests the limited impact that rehabilitation centres can have on the syndemic in the Western Cape, because of their limited capacity. One can then hypothesise that if the capacity of rehabilitation centres is significantly increased over the next five years, this intervention might show more promising results.

## 5. Discussion and Conclusion

In this study the syndemic of substance abuse and drug-related crime in the Western Cape was investigated using STELLA. The SADC-WC model was calibrated against historical data, suggesting that results and findings of the model merit exploration. Using systems thinking, the dynamic SADC-WC system allows the inclusion of the effects of the law enforcement in the system, without incorporating it into the system as a whole. This enabled us to use known data from the SAPS on drug-related cases made per year in the Western Cape.

From the simulations for the 2004–2019 system we observed that drug-related crimes will grow unceasingly if no change within the system occurs. This is clearly the case with regard to the 2015 data in which drug-related crimes increased by 2.4% [25]. Three different changes within the system were investigated as interventions: (1) assuming the cases making it to court and successful convictions will double over the next five years; (2) assuming that the rate at which correctional services refer individuals to rehabilitation centres over the next five years is doubled; and (3) assuming that there will be a decrease in the percentage of individuals that relapse back into substance abuse and drug-related crime (*D*) over the next five years. The only intervention that seemed to have a significant impact on the system was increasing the number of successful convictions. This was done, by doubling the cases that make it to court and doubling the successful conviction rate. This showed notable lower stock counts for law enforcement, in comparison with the other two suggested interventions. It was noted that focusing interventions on flows, which are governed by small stocks (in comparison to other stocks in the system), limits the impact the intervention can have on the SADC-WC system as a whole. The system dynamics model highlighted this phenomena in intervention (2) and (3), where both flows that the interventions were focused on (correctional service referrals and relapse) are governed by the dynamics of the rehabilitation stock which is notably the smallest stock in the system.

The complex dynamics of substance abuse and drug-related crime exposed in this study indicate that all the different key players (political actors, funders, program implementers, researchers, and the public) need to collaborate to counterforce this syndemic in the Western Cape. One of the challenges of complex system, like the one at hand, is that different key players often have competing goals. For instance, when communities are dependent on drug trafficking for financial income, their goal will be to expand the drug network which in turn escalates the use of substances and drug-related crime.

The results and findings of this model are only general insights that can be used as a tool to quantify and improve the understanding of the dynamics of the substance abuse and drug-related crime syndemic over time. The model highlighted the possibility that successful convictions hold with respect to encamping drug-related crime. The lack of quality and complete data for the specific focus of the model limits the credibility and accuracy of all the flows in the model. Hence the credibility and accuracy of predictions of the interventions should be taken with circumspection. We suggest expanding the SADC-WC model by creating three separate stocks to represent the syndemic, namely, one for substance users, another for drug-related criminal activity, and a stock for the individuals involved in both. We also recommend the inclusion of the judiciary as a stock and to further investigate how the flow of cases making it to court from the law enforcement influences successful convictions. This is of especial interest with regard to the observation in the model that successful convictions are a key factor in the encampment of drug-related crime. This research offers valuable insights for countries facing similar problems with substance abuse and drug-related crime such as South Africa.

## Figures and Tables

**(a):** 

Flows	Movement
Initiation	*C* → *D*
Personal conviction	*D* → *C*
Inflow	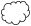 →*C*
Outflow	*C*→ 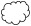
Uptake	*D* → *R*
Relapse	*R* → *D*
Cases	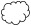 →*L*
Successful convictions	*L*→ 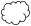
Failed convictions	*L*→ 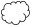
Correctional rehabilitation	*S* → *R*
Completed sentences	*S* → *C*
Successful rehabilitation	*R* → *C*
Drug- and crime-related deaths	*D*→ 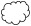
Rehabilitation deaths	*R*→ 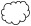
Correctional service deaths	*S*→ 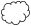

**(b):** 

Variable	Stock
*C*	Community
*D*	Drug-related crime and substance abuse
*R*	Rehabilitation
*S*	Correctional services
*L*	Law enforcement

**Table 2:** Converters and equations for the SADC-WC system, the parameters descriptions, and values are given in Table 4.

Converter	Equation
Initiation	*di∗C∗D* + *ci∗C∗D*
Self-conviction	*se∗D*
Inflow	*b∗C* + *m*
Outflow	*d∗C*
Uptake	Fixed dataset
Relapse	*r∗R*
Cases	Fixed dataset
Convictions	*c∗L*
Successful convictions	*c∗L*
Failed convictions	*fc∗L*
Correctional rehabilitation	*cc∗R*
Sentences completed	*sc∗C*
Successful rehabilitation	*sr∗R*
Drug and drug-related crime death	*dd∗D*
Correctional service death	*sd∗S*
Rehabilitation death	*rd∗R*

**Table 3:** Initial values for the SADC-WC system.

State variable	Initial value	Reference	Calculations
*L*_0_	30432	[26]	—
*R*_0_	4563	[12]	—
*C*_0_	4645600	[27]	—
*D*_0_	696840	[28]	Estimated as 15% of *D*
*S*_0_	1902	[23, 26]	Estimated as *S*(0) = *D∗*0.25*∗*.25

**Table 4:** Parameters for the SADC-WC system.

Parameter description	Symbol	Value	Reference
Birth rate	*b*	0.023	[26]
Death rate	*d*	0.013	[26]
Relapse rate	*r*	0.257	[29–31]
Correctional rehabilitation	*cc*	0.057	[29–31]
Successful conviction rate	*s*	0.250	[23]
Cases making court	*c*	0.250	[23]
Cases not making court	*fc*	0.750	[23]
Successful rehabilitation	*sr*	0.7	[29–31]
Net migration	*m*	152230	[26]
Drug initiation rate	*di*	0.05–0.1	Estimated
Crime initiation rate	*ci*	0.05–0.1	Estimated
Self-conviction rate	*se*	0.05–0.1	Estimated
Successful corrections	*sc*	0.179	[33]
Drug- and crime-related death	*dd*	0.015–0.02	Estimated
Rehabilitation related death	*rd*	0.015–0.02	Estimated
Correctional service related death	*sd*	0.015–0.02	Estimated

## Appendix

### Model Equations

*Community Compartment*

  Community(*t*) = Community(*t* − *dt*) + (Successful_Rehabilitation + Self_Conviction + Successful_Correction + Inflow − Initiation − Outflow) *∗ dt*
  INIT Community = 4645600
  INFLOWS:
  Successful_Rehabilitation = Rehabilitation *∗ sr*
  Self_Conviction = DRC_and_SA *∗ se*
  Successful_Correction = Correctional_Services *∗ sc*
  Inflow = Community*∗b* + *m*
  OUTFLOWS:
  Initiation = ((Community *∗* DRC_and_SA *∗ di*) + (Community *∗* DRC_and_SA *∗ ci*))/(Community + DRC_and_SA + Rehabilitation + Correctional_Services)
  Outflow = Community*∗d*

*Correctional Services Compartment*

  Correctional_Services(*t*) = Correctional_Services(*t* − *dt*) + (Successful_Conviction − Correctional_Rehabilitation − *S*_Death − Successful_Correction) *∗ dt*
  INIT Correctional_Services = 1902
  INFLOWS:
  Successful_Conviction = Law_Enforcement*∗s*
  OUTFLOWS:
  Correctional_Rehabilitation = Rehabilitation*∗cc*
*S*_Death = Correctional_Services*∗sd*
  Successful_Correction = Correctional_Services*∗sc*

*The DRC and SA Compartment*

  DRC_and_SA(*t*) = DRC_and_SA(*t* −* dt*) + (Initiation + Relapse − Uptake − Successful_Conviction −* D*_Death − Self_Conviction) *∗ dt*
  INIT DRC_and_SA = 696840
  INFLOWS:
  Initiation = ((Community *∗* DRC_and_SA *∗ di*) + (Community *∗* DRC_and_SA *∗ ci*))/(Community + DRC_and_SA + Rehabilitation + Correctional_Services)
  Relapse = Rehabilitation*∗r*
  OUTFLOWS:
  Uptake = GRAPH (TIME)
  (2004, 4563), (2006, 5458), (2008, 5444), (2010, 6067), (2012, 7090), (2014, 6808), (2016, 7060), (2018, 7321), (2020, 7592), (2022, 7873), (2024, 8164)
  Successful_Conviction = Law_Enforcement*∗s*
*D*_Death = DRC_and_SA *∗ dd*
  Self_Conviction = DRC_and_SA *∗ se*

*Law Enforcement*

  Law_Enforcement(*t*) = Law_Enforcement(*t* − *dt*) + (Cases − Failed_Convictions − Succesful_Convictions) *∗ dt*
  INIT Law_Enforcement = 30432
  INFLOWS:
  Cases = GRAPH(TIME)
  (2004, 19940), (2006, 34788), (2008, 45985), (2010, 60409), (2012, 76650), (2014, 84337), (2016, 92771), (2018, 102048), (2020, 112253), (2022, 123478), (2024, 135825)
  OUTFLOWS:
  Failed_Convictions = Law_Enforcement *∗ fc*
  Succesful_Convictions = Law_Enforcement *∗ s ∗ c*

*Rehabilitation*

  Rehabilitation(*t*) = Rehabilitation(*t* − *dt*) + (Uptake + Correctional_Rehabilitation − Successful_Rehabilitation − *R*_Death − Relapse) *∗ dt*
  INIT Rehabilitation = 4563
  INFLOWS:
  Uptake = GRAPH(TIME)
  (2004, 4563), (2006, 5458), (2008, 5444), (2010, 6067), (2012, 7090), (2014, 6808), (2016, 7060), (2018, 7321), (2020, 7592), (2022, 7873), (2024, 8164)
  Correctional_Rehabilitation = Rehabilitation*∗cc*
  OUTFLOWS:
  Successful_Rehabilitation = Rehabilitation*∗sr*
*R*_Death = Rehabilitation*∗rd*
  Relapse = Rehabilitation*∗r*
*b* = .023
*c* = 0.25
*cc* = 0.057
*ci* = GRAPH(TIME)
  (2004, 0.0598), (2006, 0.0614), (2008, 0.0625), (2010, 0.0657), (2012, 0.0708), (2014, 0.076), (2016, 0.079), (2018, 0.0811), (2020, 0.0835), (2022, 0.0868), (2024, 0.0908)
*d* = 0.013
*dd* = GRAPH(TIME)
  (2004, 0.0175), (2006, 0.0192), (2008, 0.0194), (2010, 0.018), (2012, 0.018), (2014, 0.0195), (2016, 0.0194), (2018, 0.0176), (2020, 0.0185), (2022, 0.0195), (2024, 0.0192)
*di* = GRAPH(TIME)
  (2004, 0.0613), (2006, 0.0638), (2008, 0.0667), (2010, 0.0694), (2012, 0.0724), (2014, 0.0749), (2016, 0.0789), (2018, 0.0824), (2020, 0.0849), (2022, 0.0879), (2024, 0.0946)
*fc* = 0.75
*m* = 152230
*r* = 0.257
*rd* = GRAPH(TIME)
  (2004, 0.016), (2006, 0.0178), (2008, 0.0187), (2010, 0.0188), (2012, 0.018), (2014, 0.0161), (2016, 0.0161), (2018, 0.0169), (2020, 0.0186), (2022, 0.0188), (2024, 0.0186)
*s* = 0.25
*sc* = (1/2.5*∗*0.31 + 1 − 10 + 0.42 + 1/12.5*∗*0.10 + 1/30*∗*0.16)
*sd* = GRAPH(TIME)
  (2004, 0.019), (2006, 0.0191), (2008, 0.0188), (2010, 0.0169), (2012, 0.0163), (2014, 0.0164), (2016, 0.0192), (2018, 0.019), (2020, 0.0179), (2022, 0.0165), (2024, 0.0166)
*se* = GRAPH(TIME)
  (2004, 0.0152), (2006, 0.0358), (2008, 0.0376), (2010, 0.0171), (2012, 0.0416), (2014, 0.043), (2016, 0.0382), (2018, 0.0194), (2020, 0.0341), (2022, 0.0363), (2024, 0.0198)
*sr* = 0.7
